# Epithelial cell-derived cytokines CST3 and GDF15 as potential therapeutics for pulmonary fibrosis

**DOI:** 10.1038/s41419-018-0530-0

**Published:** 2018-05-03

**Authors:** Young-Im Kim, Hyun-Woo Shin, Yang-Sook Chun, Chung-Hyun Cho, Jaemoon Koh, Doo Hyun Chung, Jong-Wan Park

**Affiliations:** 1 Department of Biomedical Sciences, BK21-plus Education Program, Seoul National University College of Medicine, Seoul, Korea; 2 Department of Pharmacology, Seoul National University College of Medicine, Seoul, Korea; 3 Ischemic/Hypoxic Disease Institute, Seoul National University College of Medicine, Seoul, Korea; 4 Department of Pathology, Seoul National University Hospital, Seoul, Korea

## Abstract

While wound healing is completed, the epithelium functions to normalize the interstitial context by eliminating fibroblasts excited during matrix reconstruction. If not, tissues undergo pathologic fibrosis. Pulmonary fibrosis is a fatal and hardly curable disorder. We here tried to identify epithelium-derived cytokines capable of ameliorating pulmonary fibrosis. Human lung fibroblasts were inactivated in epithelial cell-conditioned media. Cystatin C (CST3) and growth differentiation factor 15 (GDF15) were found to be enriched in the conditioned media and to inhibit the growth and activation of lung fibroblasts by inactivating the TGF–Smad pathway. In mouse and human lungs with interstitial fibrosis, CST3 and GDF15 expressions were markedly reduced, and the restoration of these cytokines alleviated the fibrotic changes in mouse lungs. These results suggest that CST3 and GDF15 are bona fide regulators to prevent excessive proliferation and activation of fibroblasts in injured lungs. These cytokines could be potential therapeutics for ameliorating interstitial lung fibrosis.

## Key points

- Epithelial cell-derived CST3 and GDF15 are fibroblast-inhibiting cytokines
- CST3 and GDF15 inhibit the TGF-signaling pathway in fibroblasts
- CST3 and GDF15 in the lung are downregulated during fibrosis
- Recombinant CST3 and GDF15 ameliorate pulmonary fibrosis in vivo

## Introduction

Pulmonary fibrosis is a chronic progressive lung disorder associated with excessive extracellular matrix (ECM) deposition and collapse of the lung parenchymal architecture, leading to severe respiratory dysfunction with a median survival of 2–4 years^1^. Anti-inflammatory and immunosuppressive drugs have been tested as therapeutic regimens for pulmonary fibrosis, but none have been sufficiently effective in prolonging the survival period of patients^2^. Based on a consensus that pulmonary fibrosis is attributed to an overgrowth of activated fibroblasts^3^, anti-fibrotic agents have been tried as emerging drugs for treating pulmonary fibrosis. Indeed, nintedanib and pirfenidone were clinically tried and evaluated to delay the progression of fibrosis^4,5^. However, these drugs were reported to provoke serious adverse effects in the clinical trial^6–8^.

In most tissues, epithelial–mesenchymal homeostasis must be maintained for normal structures and functions^9^. For appropriate recovery of injured epithelium, the wound healing process must complete three steps—inflammation, proliferation, and maturation phases. Finally, for the recovery of epithelial–mesenchymal homeostasis, outgrown fibroblasts should be eliminated from the repaired tissue^10^. Currently, pulmonary fibrosis is understood as a disorder of epithelial–mesenchymal homeostasis because the epithelial integrity fail to be repaired during repeated injury–regeneration. Consequently, the wound healing process cannot be halted and the fibroblast stimulation continues, because^11^. Furthermore, myofibroblasts induce epithelial cell death and disturb the epithelial repair process. In fibrotic tissue, injured epithelial cells and outgrown myofibroblasts activate a positive feedback loop that results in massive fibrosis and alveolar destruction^12^.

Cystatin C (CST3) is a cytokine ubiquitously expressed in most mammalian cells and also detected in blood and body fluids^13^. Given that it potently inhibits cysteine proteases like cathepsins, CST3 is expected to stimulate fibrosis by inhibiting the protease-mediated digestion of ECM^14,15^. In contrast, cathepsins have been also reported to promote liver or lung fibrosis by facilitating TGF-β-driven differentiation of fibroblasts^16,17^. To date, the roles of cathepsins and CST3 in organ fibrosis are controversial. On the other hand, growth differentiation factor 15 (GDF15) is a TGF-β family member that is induced immediately after a harmful stress^18^. GDF15 is believed to be associated with stress responses, but its biological functions have not been clearly identified. Although GDF15 has been shown to promote cancer cell death, whether it controls fibroblast proliferation and activation is unclear^18–20^.

Despite many efforts to understand the pathogenesis of pulmonary fibrosis, little is known about the mechanism of epithelial cell control over fibroblasts in maintaining epithelial–mesenchymal homeostasis. Identifying fibroblast-controlling cytokines could provide novel peptide drugs for pulmonary fibrosis therapy. In this study, we identified two epithelial cell-derived cytokines CST3 and GDF15 capable of inhibiting proliferation and activation of fibroblasts. Furthermore, we tested the ability of the cytokines to ameliorate bleomycin-induced pulmonary fibrosis in mice.

## Results

### Lung fibroblast growth is inhibited in alveolar epithelial cell-conditioned media

**Fig. 1:**
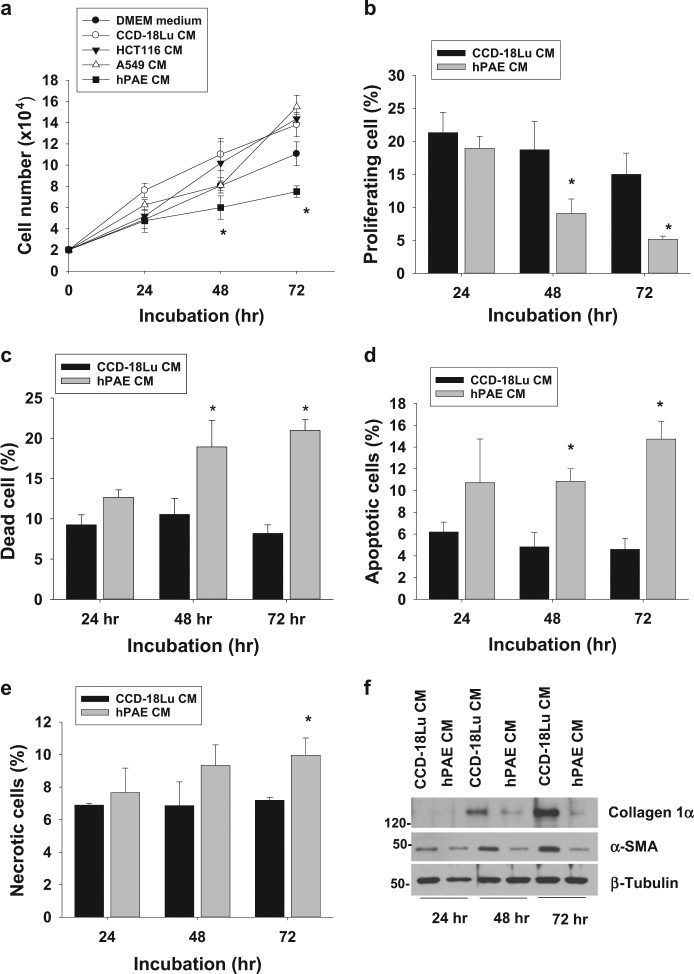
Lung fibroblast growth is inhibited in the conditioned medium from pulmonary alveolar epithelial cells. **a** CCD-18Lu cells were incubated in conditioned media (CM) collected from the indicated cells. Viable CCD-18Lu cells (unstained with trypan blue) were counted using a hemocytometer. **b** After CCD-18Lu cells were incubated in the indicated CMs for 1–3 days, the population of proliferating cells was determined by counting BrdU-stained cells at the S phase on flow cytometry. **c** CCD-18Lu cells were incubated in the indicated CM, and dead cells (stained with trypan blue) were counted using a hemocytometer. **d**, **e** CCD-18Lu cells were co-stained with annexin V-FITC for apoptosis and propidium iodide for necrosis, and subjected to flow cytometry. **f** CCD-18Lu cells were incubated in the indicated CMs for 1–3 days. After floating cells were removed, attached cells were subjected to immunoblotting. Data in all panels are presented as the means and s.d. (*n* = 3). *P < 0.05 vs. the CCD-18Lu CM group. Statistics: Student *t*-test in (**a**, **e**) panels; one-way ANOVA with Post hoc Tukey test in (**b**–**d**)

To determine which cells produced fibroblast-inhibiting factors, we incubated lung fibroblast cell lines CCD-18Lu in a mixture (1:1) of a fresh medium and a conditioned medium (CM) collected from various epithelium-derived cells, including human pulmonary alveolar epithelial cells (hPAE) and 2 carcinoma (A549 and HCT116) cell lines. Before collecting conditioned media, we verified that all cells could maintain their viabilities in serum-free DMEM medium (data not shown). The growth of CCD-18Lu cells was significantly attenuated in hPAE CM (Fig. 1a). To understand the properties of fibroblast growth arrest, proliferating, dead, apoptotic, or necrotic cells were counted. Given that BrdU-positive cell in the S phase were regarded as proliferative (Figure S1a), cell proliferation was halted in hPAE CM (Fig. 1b). To analyze cell death, trypan blue-stained cells were counted, which indicates that cells undergo death in hPAE CM (Fig. 1c). We evaluates the types of cell death by co-staining cells with annexin V and propidium iodide (Figure S1b), and found that hPAE CM provoked either apoptosis or necrosis in CCD-18Lu cells (Fig. 1d,e). To confirm apoptosis, cleaved caspase 3 and PARP were detected by Western blotting. Both apoptosis markers increased in CCD-18Lu cells incubated with hPAE CM (Figure S1c). We next analyzed the cellular levels of α-smooth muscle actin (α-SMA) and collagen-1α, which are representative markers of active myofibroblasts^10,21^. Given that both extracellular proteins were upregulated incubation period-dependently, CCD-18Lu cells were spontaneously activated under fetal bovine serum and CM. However, this fibroblast activation did not occur in hPAE CM (Fig. 1f). Based on the results, normal epithelial cells are likely to release some factors against fibroblast growth and activation.

### Alveolar epithelial cell-derived CST3 and GDF15 inhibit lung fibroblast growth

**Fig. 2:**
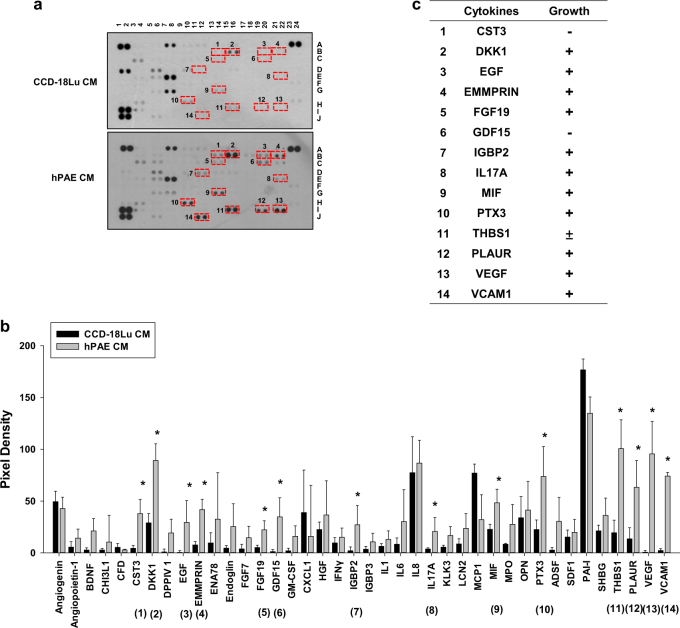
Identification of cytokines secreted from hPAE cells. **a** The conditioned media collected from CCD-18Lu and hPAE cells were applied to the proteome profiling arrays and proteins were visualized using ECL. Red boxes indicate the cytokines whose levels are relatively higher in hPAE-conditioned media compared to CCD-18Lu. **b** The intensities of dots in the arrays were quantified using the ImageJ program. The pixel densities are presented as bars (means + s.d., *n* = 3) and *P < 0.05 vs. both CCD-18Lu CM by Student *t*-test. **c** The cytokines enriched in hPAE-conditioned media are listed. In the growth category, −, +, and ± represent negative, positive, and controversial effects on cell growth, respectively

**Fig. 3:**
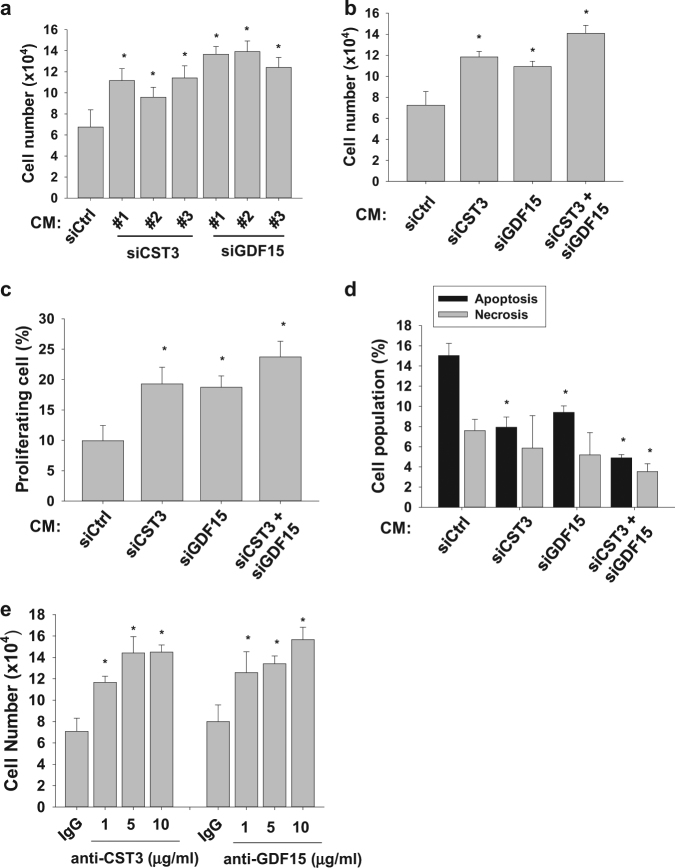
CST3 and GDF15 are responsible for fibroblast inhibition by hPAE CM. **a** CCD-18Lu cells were incubated for 48 h in CM from hPAE cells transfected with the indicated siRNAs (40 nM). Three different siRNAs (#1, #2, and #3) for CST3 or GDF15 were tested. Viable CCD-18Lu cells were counted (right). **b**,**c** hPAE cells were transfected with 20 nM siRNA targeting CST3 or GDF15, or with both (20 nM each). After 48 h, cell growth rate were measured by hematocytometer, and the population of BrdU-stained CCD-18Lu cells at S phase were analyzed by flow cytometry. **d** Apoptotic and necrotic CCD-18Lu cells were marked with annexin V-FITC and propidium iodide and subjected to flow cytometry. **e** hPAE CM was pre-incubated with non-immunized serum (IgG), anti-CST3, or anti-GDF15 antibody at the indicated doses for 1 h. CCD-18Lu cells were incubated for 48 h in the conditioned media, and viable cells were counted. Each bar in all panels represents the mean + s.d. (*n* = 3). *P < 0.05 vs. the si-Ctrl group or the IgG group. Statistics: Student's *t*-test in (**a**) and (**e**) panels; one-way ANOVA with post hoc Tukey test in (**b**–**d**)

To examine whether the fibroblast-inhibiting factors are composed of polypeptides, hPAE CM was heated to denature polypeptides or trypsinized to degrade them. After heating or trypsinizing, hPAE CM almost completely lost its ability to inhibit fibroblast growth (data not shown). These results strongly indicate that the fibroblast-inhibiting factors are polypeptides. To identify peptide-based cytokines in CMs, CCD-18Lu and hPAE CMs were tested with the Human XL Cytokine Array Kit. The red boxes (1–14) indicate cytokines that were more abundant in hPAE CM than in CCD-18Lu. (Fig. 2a). Cytokine levels quantified from pixels in the ImageJ program are presented as bar graphs (Fig. 2b). The cytokines enriched in hPAE CM are summarized in Fig. 2c. After reviewing the literatures on cytokine functions, we determined two candidates as fibroblast-inhibiting cytokines: cystatin C (CST3) has been reported to inhibit fibrosis by antagonizing TGF-β;^22,23^ growth differentiation factor 15 (GDF15) to induce cancer cell death^18,20^. In immunoblot analyses, CST3 and GDF15 were identified to accumulate over time in hPAE CM. (Figure S2a). To examine whether the cytokines inhibit fibroblast growth, we prepared CMs from hPAE cells in which each cytokine was knocked-down. To rule out off-target effects of siRNAs, we assessed and confirmed the fibroblast-inhibiting effects of CST3 and GDF15 siRNAs targeting different sites of each mRNA (Fig. 3a and S2b). These siRNAs per se remaining in the CMs did not affect the CCD-18Lu growth (data not shown). When both cytokines were knocked-down together, cell proliferation and survival both were additively enhanced (Fig. 3b–d, and S3). To confirm the roles of these cytokines in fibroblast inhibition, we incubated CCD-18Lu cells in hPAE CM pretreated with a neutralizing antibody against CST3 or GDF15 for 48 h. Each antibody rescued fibroblast growth even in hPAE CM (Fig. 3e). These results suggest that CST3 and GDF15 are secreted from normal alveolar epithelial cells and are involved in the inhibition of fibroblast growth.

### Recombinant CST3 and GDF15 inhibit fibroblast growth and activity in cell culture

**Fig. 4:**
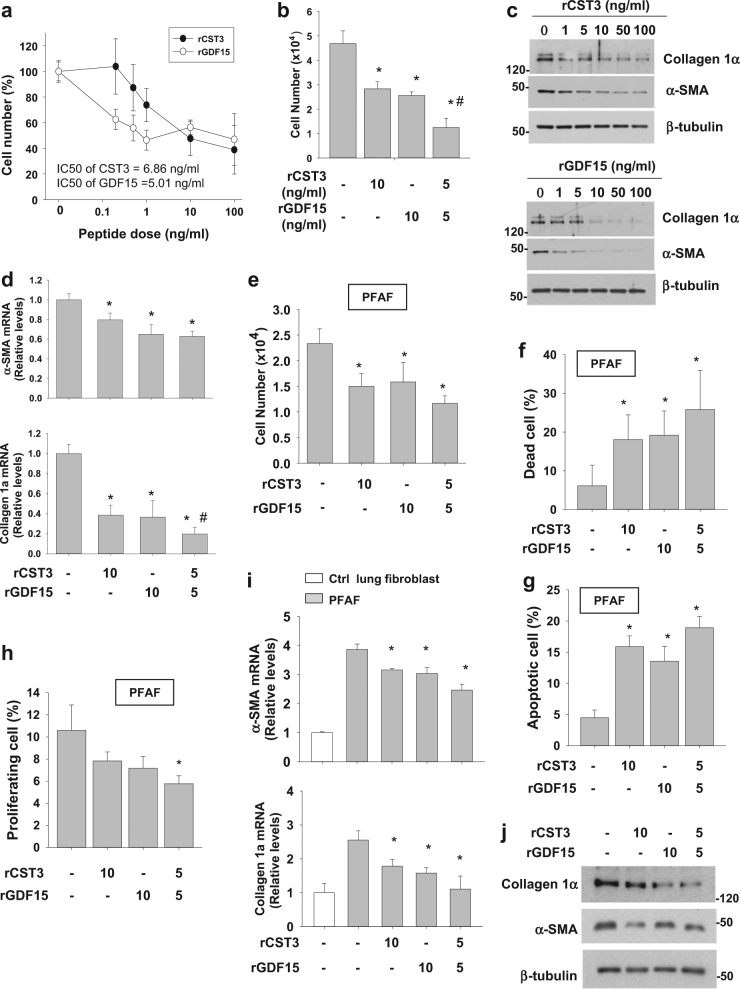
CST3 and GDF15 peptides inhibit proliferation and ECM production in CCD-18Lu cells. **a** CCD-18Lu cells were treated with a recombinant peptide of CST3 or GDF15 and cultured in 2% serum-containing medium for 24 h. IC50s are the doses of peptides showing 50% growth inhibition. **b** CCD-18Lu cells were incubated with one of two peptides (10 ng/mL) or both peptides (5 ng/mL each) for 48 h. **c** CCD-18Lu cells were incubated with CST3 or GDF15 peptide for 48 h, and subjected to immunoblotting. **d** CCD-18Lu cells were incubated with one of two peptides (10 ng/mL) or both peptides (5 ng/mL each) for 24 h. The mRNA levels were measured by RT-qPCR. All data are presented as the means + s.d. from three experiments. *P < 0.05 vs. the PBS group; ^#^*P* < 0.05 vs. the CST3 or GDF15 only group. **e**,**f** Primary lung fibroblasts were isolated from fibrotic tissues of bleomycin-treated mouse lungs, and cultured. The pulmonary fibrosis-associated fibroblasts (PFAFs) were pre-incubated in a 2% FBS-containing medium for 16 h, and then treated with CST3 or/and GDF15 (5 or 10 ng/mL) for 24 h. Viable (unstained, **e**) and dead (stained, **f**) cells were counted using a hemocytometer. **g**,**h** PFAFs, which had been treated with CST3 or/and GDF15 for 24 h, were co-stained with annexin V-FITC and propidium iodide (**g**) or with co-stained with BrdU and 7-AAD (**h**), and subjected to flow cytometry. **i** Control lung fibroblasts were treated with PBS, and PFAFs were treated with PBS or CST3 or/and GDF15 in 2% FBS-containing media for 24 h. The mRNA levels were measured by RT-qPCR. **j** PFAFs were treated with CST3 or/and GDF15 for 24 h, lysed, and subjected to immunoblotting. All data are presented as the means + s.d. from three experiments.*P < 0.05 vs. the untreated PFAF group. Statistics: Student's *t*-test in (**a**); one-way ANOVA with post hoc Tukey test in (**b**–**i**)

To confirm the actions of CST3 and GDF15 against fibroblast growth, CCD-18Lu was treated with recombinant peptides of human CST3 (rCST3) and human GDF15 (rGDF15), and cell numbers were counted 24 h after the treatment. Both peptides reduced the number of CCD-18Lu cells in a concentration-dependent manner (Fig. 4a). Based on the IC_50_ (half maximal inhibitory concentration) values, rGDF15 and rCST3 seem to have the similar efficacy. In contrast, the peptides failed to inhibit cell growth in hPAE cells (Figure S8a), supporting the fibroblast-specific actions of these peptides. To test the synergistic effects of rCST3 and rGDF15, we compared the fibroblast-inhibiting effects of 1 ng/mL of each peptide with a combination of half-concentrations (0.5 + 0.5 ng/mL) of the two peptides. The combination showed a greater (>twofold) effect than the single treatment of each peptide (Fig. 4b). Given that the combination index (CI) was <1.0 on the compusyn software (CompuSyn Inc.; Paramus, NJ)^24^, the combination of CST3 and GDF15 seems to have a synergistic effect on fibroblast inhibition. Aside from growth inhibition, both peptides downregulated collagen-1α and α-SMA protein and mRNA levels (Fig. 4c,d). In addition, we isolated pulmonary fibrosis-associated fibrosis (PFAF) from mouse lungs with bleomycin-induced fibrosis to observe responses of activated lung fibroblasts. rGDF15 or/and rCST3 diminished PFAF numbers (Fig. 4e), promoted cell death (Fig. 4f,g), and reduced proliferation potential (Fig. 4h). rGDF15 or/and rCST3 also downregulated collagen-1α and α-SMA protein and mRNA levels (Fig. 4i,j). These results encouraged us to investigate whether the peptides are applicable to prevent pulmonary fibrosis.

### CST3 and GDF15 inhibit the TGF-signaling pathway

**Fig. 5:**
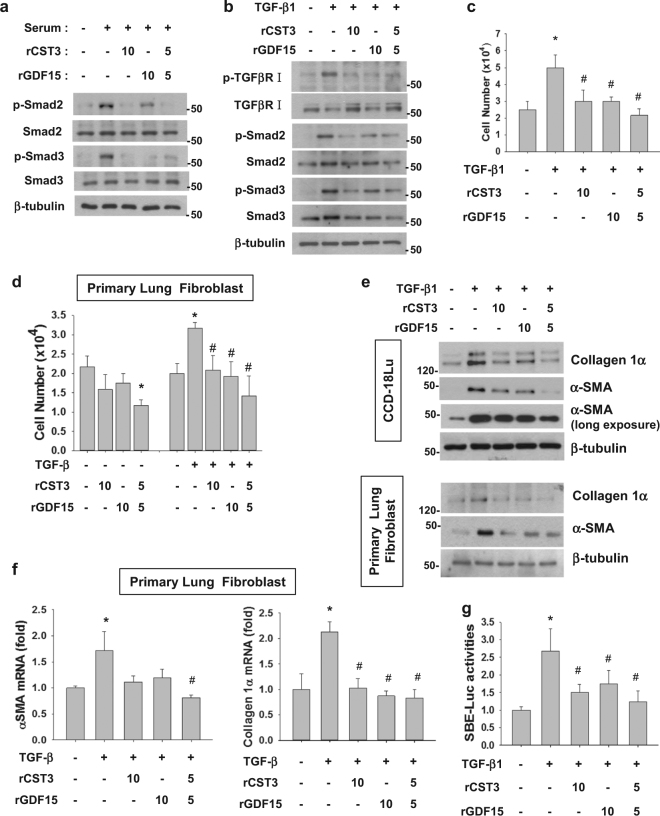
CST3 and GDF15 inhibit the TGF-β signaling pathway in CCD-18Lu cells. **a**,**b** CCD-18Lu cells, which had been pre-incubated in serum-free media for 16 h, were treated with a recombinant peptide (10 ng/mL) of CST3 or GDF15 in the presence of 5 % FBS (**a**) or 5 ng/mL TGF-β1 (**b**) for 1 h, and subjected to immunoblotting. **c** CCD-18Lu cells were pre-incubated in a serum-free medium for 16 h, and then treated with TGF-β1 and CST3 or/and GDF15 for 24 h. CCD-18Lu cells were counted using a hemocytometer. **d** Primary lung fibroblasts, which had been isolated from normal mouse lung, were pre-incubated in a serum-free medium for 16 h, and then treated with TGF-β1 and CST3 or/and GDF15 for 24 h. CCD-18Lu cells were counted using a hemocytometer. **e** CCD-18Lu cells or primary lung fibroblasts were treated with TGF-β1 and CST3 or/and GDF15 for 24 h, and subjected to immunoblotting. **f** Primary lung fibroblasts were treated with TGF-β1 and CST3 or/and GDF15 for 24 h. The mRNA levels were measured by RT-qPCR. **g** CCD-18Lu cells, which had been co-transfected with SBE-Luc and β-galactosidase plasmids, were treated with TGF-β1 and CST3 or/and GDF15 for 24 h. The luciferase activity was divided by β-galactosidase activity to normalize transfection efficiency. All data are presented as the means + s.d. from three experiments. *P < 0.05 vs. the untreated group; ^#^*P* < 0.05 vs. the TGF-β1 only group by one-way ANOVA with post hoc Tukey test

Given that fibroblast growth and activation largely depend on the TGF–Smad pathway, we first checked whether CST3 and GDF15 inhibit the signaling pathway. As expected, Smad2/3 in CCD-18Lu were phosphorylated (or activated) in the presence of FBS. Interestingly, the phosphorylation was attenuated by recombinant CST3 or/and GDF15 (Fig. 5a). When the TGF-signaling pathway was provoked by TGF-β1, TGF-β receptor, and Smad2/3 were inactivated by CST3 or/and GDF15 (Fig. 5b). In either CCD-18Lu cells (Fig. 5c) or primary mouse lung fibroblasts (Fig. 5d), cell growth was stimulated by TGF-β1, which was abolished by the recombinant peptides. ECM production in CCL-18Lu and mouse lung fibroblast was also stimulated by TGF-β1, but inhibited by the recombinant peptides (Fig. 5e). Collagen 1α and αSMA expressions in mouse lung fibroblasts were repressed at the transcriptional level by the peptides (Fig. 5f). We next evaluated the TGF/Smad-dependent gene expression using the SBE-luciferase reporter, and confirmed the inhibitory actions of CST3 and GDF15 to the TGF–Smad signaling pathway (Fig. 5g).

### CST3 and GDF15 are downregulated in mouse and human lungs undergoing fibrosis

**Fig. 6:**
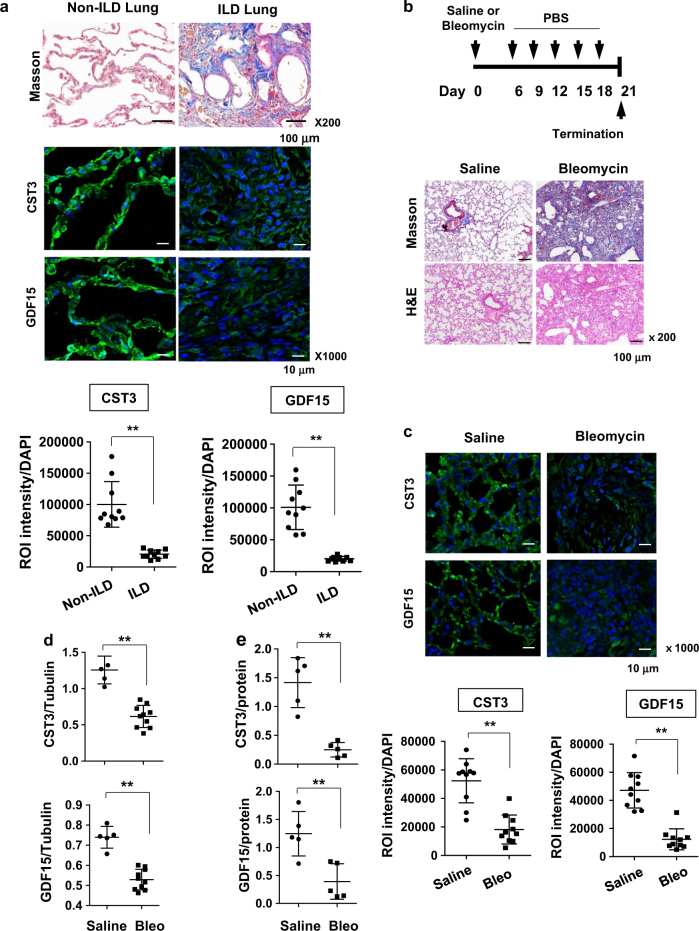
CST3 and GDF15 are downregulated in mouse and human lungs with fibrosis. **a** Tissue specimens of non-ILD (*n* = 10) and ILD (*n* = 10) human lungs were subjected to Masson trichrome staining (top, ×200 magnification) and immunofluorescence staining with anti-CST3 or anti-GDF15 antibodies (middle, ×1000 magnification). The immune complexes and DAPI-stained nuclei were visualized under a fluorescence microscope. Total fluorescence intensity was quantified using ImageJ and divided by the number of nuclei. Data are plotted as dots (bottom). **b** Experimental schedule for bleomycin-induced pulmonary fibrosis (top). Saline-treated or bleomycin-treated lung sections were stained with Masson trichrome and H&E (bottom). **c** Tissue specimens of saline-treated or bleomycin-treated mouse lungs (10 in each group) were subjected to immunofluorescence staining with anti-CST3 or anti-GDF15 antibodies (top). Total fluorescence intensity was quantified using ImageJ and divided by the number of nuclei (bottom). **d** CST3 and GDF15 proteins were immunoblotted in mouse lung homogenates prepared from saline-treated or bleomycin-treated mice for 21 days, and their levels vs. β-tubulins were quantified using ImageJ. **e** Bronchoalveolar lavage fluid were collected from saline-treated or bleomycin-treated mice for 21 days, and proteins in the fluid were precipitated by TCA. CST3 and GDF15 levels were detected by Western blotting, and their levels vs. protein levels were quantified using ImageJ. The means and s.d. in dot plots are presented by long and short horizontal bars and **P < 0.01 by Student's *t*-test

At the last step of wound repair, the epithelium may function to normalize the ECM microenvironment by pacifying fibroblasts excited during ECM reconstruction. Yet, the epithelium-derived, fibroblast-controlling factors have not been identified. Our results prompted us to test the possibility that CST3 and GDF15 are bona fide controller of fibroblasts in the lung. First, we analyzed the expression of CST3 and GDF15 in lung tissues of patients with and without interstitial lung disease (ILD). Masson’s trichrome staining verified that the lung tissues from ILD patients were filled with massive ECM but those from lung cancer patients without ILD had normal architecture. CST3 and GDF15 were observed to be highly expressed along the alveolar wall in non-ILD lungs. Surprisingly, both cytokines were barely present in ILD lungs (Fig. 6a, top). All histological data in human lung specimens are shown in Figures S7-10. To quantify the cytokine levels, fluorescence intensities were normalized to cell numbers. The expressions of both cytokines were significantly lower in ILD lungs than in non-ILD lungs (Fig. 6a, bottom). To confirm the suppression of the cytokines during fibrosis, we adopted an animal model for pulmonary fibrosis. C57/B6 mice were subjected to a single challenge of bleomycin via bronchial instillation, and intraperitoneally injected twice weekly with PBS. On Day 21 after the bleomycin treatment, lung tissues were prepared for histological analyses. Figure 6b shows the experimental schedule and the results from Masson’s trichrome staining. The procedure using bleomycin successfully induced excessive deposition of collagen in the interstitium of mouse lungs. As was shown in ILD lungs, both the cytokines were markedly suppressed in fibrotic lungs of mice, which was double-checked by immunofluorescence analysis (Fig. 6c) and immunoblotting (Fig. 6d, figure S11a). To examine whether these cytokines were released from alveolar epithelium, we measured CST3 and GDF15 in BALF, and found that CST3 and GDF15 levels were substantially reduced in fibrotic lungs (Fig. 6e and S11b). Accordingly, the suppression of these cytokines in fibrotic lungs provides a rationale to restore the cytokines for treating lung fibrosis.

### Recombinant CST3 and GDF15 ameliorate pulmonary fibrosis in vivo

**Fig. 7:**
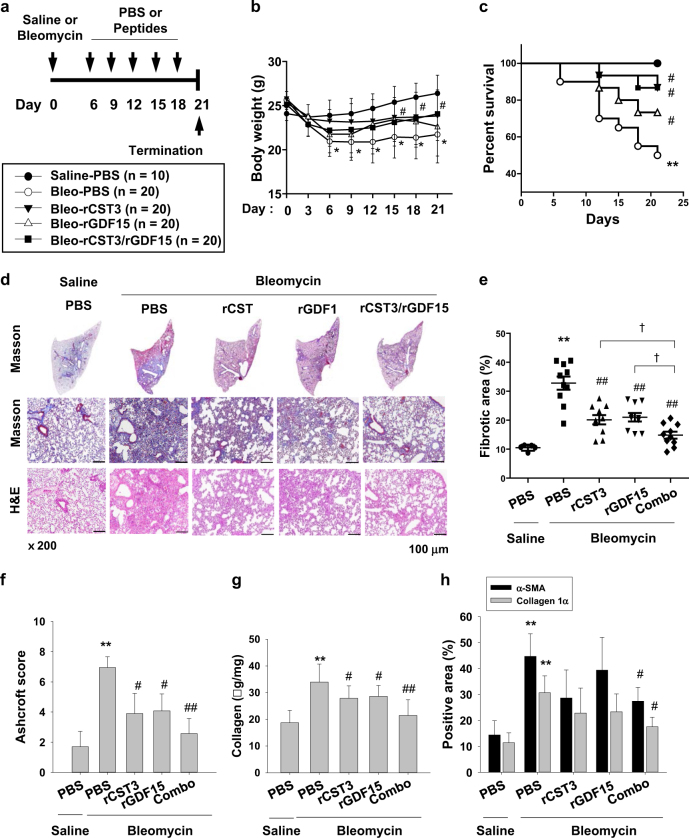
Recombinant CST3 and GDF15 ameliorate lung fibrosis in bleomycin-treated mice. **a** Experimental schedule for pulmonary fibrosis and peptide injection and the numbers of mice used. **b** Mouse body weights (means and s.d.). **c** Kaplan–Meier survival curves. Survival rates in Bleo-rCST3 and Bleo-rCST3/rGDF15 groups were higher significantly than in Bleo-PBS group. **d** Saline-treated or bleomycin-treated lungs and tissue sections were stained with Masson trichrome or H&E. **e** Masson trichrome staining was quantified using ImageJ. Long and short horizontal bars represent the means and s.d. **f** Ashcroft scores were analyzed in Masson trichrome-stained sections. **g** Hydroxyproline contents in lung homogenates were quantified spectrophotometrically and collagen expression were determined by lung weight. Statistics was analyzed using one-way ANOVA with post hoc Tukey test. **h** α-SMA and collagen-1α in lung tissues were immunohistochemically stained, and quantified using ImageJ. Each point or bar in all panels represents the mean + s.d. *P < 0.05 and **P < 0.01 vs. the control group, respectively; ^#^*P* < 0.05 and ^##^*P* < 0.01 vs. the Bleo-PBS group; ^†^*P* < 0.05 between the indicated groups. Statistics: one-way ANOVA with Post hoc Tukey test in (**b**), (**e**), (**g**), and (**h**) panels; Log-rank test in (**c**); Mann–Whitney *U* test in (**f**)

After a bronchial instillation of bleomycin, C57/B6 mice were systemically injected twice weekly with PBS, rCST3 (100 μg/kg), rGDF15 (100 μg/kg), or both peptides (50 μg/kg of each). On the 21st day, lung tissues were excised, as illustrated in Fig. 7a. Body weight was measured continuously to monitor apparent health. Mouse weights gradually decreased over the 21 days, but was significantly recovered by combined peptides (Fig. 7b). Mouse survival was also improved by the combination (Fig. 7c). To evaluate the extent of pulmonary fibrosis, we stained lung specimens with Masson’s trichrome and hematoxylin/eosin (Fig. 7d). As expected, bleomycin induced an obstruction of the alveolar sacs with thickened inter-alveolar septa and massive collagen in the lung interstitium. Administration of either rCST3 or rGDF15 saved airways spaces and lessened fibrosis in bleomycin-treated lungs, and the combination of these peptides at the half doses was more effective than any single administration (Fig. 7d). To quantify the fibrotic changes, we measured fibrotic areas and Ashcroft scores on Masson’s trichrome-stained specimens, and biochemically analyzed hydroxyproline levels in lung tissues (Fig. 7e–g). The results of these fibrosis parameters further supported our hypothesis that rCST3 and rGDF15 in combination attenuates bleomycin-induced pulmonary fibrosis. Next, we analyzed α-SMA and collagen-1α levels to evaluate the degree of fibroblast activation. Both markers were densely stained in the lungs treated with bleomycin, but were less obvious in peptide-treated lungs (Fig. 7h, S12). Collectively, recombinant CST3 and GDF15 could be developed as potential biopharmaceuticals for treating pulmonary fibrosis.

## Discussion

Under the assumption that inflammation provokes fibrosis, anti-inflammatory medications have been tested as pulmonary fibrosis therapeutics, which include corticosteroids, azathioprine, chlorambucil, cyclophosphamide, cyclosporine, and interferons^25^. However, these therapeutics failed to reduce the mortality rate of pulmonary fibrosis in clinical trials and induced side effects^26,27^. More recently, agents with diverse pharmacological actions, including antioxidation and anticoagulation, have been tested clinically, but have not led to sufficient improvements^28,29^. The poor outcomes of anti-inflammatory therapies caused the change in the assumption of pulmonary fibrosis pathogenesis. Pulmonary fibrosis is now believed to result from aberrant wound repair accompanying the hyperactivation of interstitial fibroblasts and disorganization of the alveolar epithelium^30^. Based on this concept, fibroblast-targeting therapies are becoming a popular focus of research as a new strategy to treat pulmonary fibrosis. For instance, pirfenidone, tyrosine-kinase inhibitors, TGF-β inhibitors, and connective tissue growth factor inhibitors have been evaluated in phase II and III clinical trials^31,32^.

Fibroblast-targeting pulmonary fibrosis therapies can be mainly classified as small molecules or monoclonal antibodies. Since signaling pathways stimulating fibroblasts have been considered as the primary targets, investigations might focus on chemical inhibitors or antibodies that can block fibroblast-activating pathways^33,34^. However, we here shifted the anti-fibrosis strategy to reinforcing fibroblast-suppressive signaling pathways. According to the epithelial–mesenchymal homeostasis theory, the pulmonary epithelium has the intrinsic ability to suppress active fibroblasts after wound repair completion. This inspired our research for new pulmonary fibrosis therapies. We discovered two fibroblast-inhibiting cytokines, CST3 and GDF15, from normal epithelial cells and demonstrated their therapeutic effects in a bleomycin-induced pulmonary fibrosis mouse model.

Bleomycin-induced pulmonary fibrosis is common in animal models to test potential pulmonary fibrosis therapeutics. Bleomycin acutely simulates pulmonary inflammation, followed by chronic progression of pulmonary fibrosis. As molecular signatures of fibrosis, pro-inflammatory cytokines, such as IL-1, IL-6, and TNF-α, increase at the early phase in lung tissues treated with bleomycin^35^. Later, pro-fibrotic markers, such as fibronectin, procollagen-1, and TGF-β, gradually increase in the tissues and their levels peak around 2 weeks after bleomycin treatment^36,37^. Actually, therapy during the acute inflammatory phase may not be practical in hospitals, as pulmonary fibrosis can be diagnosed after onset of respiratory symptoms and detection of radiological evidence. Therefore, therapy might be started during the fibrotic phase. Considering the practical period of therapy, we started to inject the recombinant peptides 6 days after the bleomycin treatment. Our preliminary experiment indicated that the lung tissues partially underwent fibrosis on day 6, which helped us to determine the schedule for injecting peptides. Even though pulmonary fibrosis had already started, the peptides were sufficiently effective as to halt the disease progression (Fig. 7). Therefore, cytokine therapy is expected to be effective in patients who are already diagnosed with pulmonary fibrosis.

Given that cathepsins can digest ECM proteins, cathepsins is expected to reduce fibrotic burden. However, the fibrosis-promoting effects of cathepsins have been shown in several literatures. For instance, cathepsin B stimulates hepatic stellate cells to promote fibrogenesis^14^, and inhibiting cathepsin B suppresses collagen deposition in mouse livers^15^. Cathepsin B also stimulates fibrogenesis by facilitating TGF-β-driven differentiation of lung fibroblasts^16,17^. Therefore, it is reasonable that CST3 ameliorates lung fibrosis by inhibiting cathepsins. Independent of cathepsin inhibition, CST3 is known to block fibrogenesis by directly antagonizing the TGF-β pathway. It physically binds to the TGF-β receptor, inhibiting TGF-β from interacting with its receptor^16,22^. Our results showing that CST3 attenuates the TGF signaling in CCD-18Lu support the latter mechanism of CST3. However, we cannot rule out the possibility that CST3 prevents fibrogenesis in bleomycin-treated lungs of mice via the dual effects of CST3 against lung fibroblasts.

The biological roles of GDF15 have been investigated restrictively in cancer cells. GDF15 is known to induce apoptosis in colorectal, prostate, and lung cancer cells^18–20^. Since the GDF15 gene harbors the p53 response element in the promoter region^38^, GDF15 is also expected to induce fibroblast death under harmful conditions like hypoxia and inflammation. However, GDF15 was unexpectedly found to inhibit fibroblast growth and activation through the inhibition of the TGF–Smad pathway. In contrast to these results, a recent report demonstrated that GDF15 stimulated proliferation of NIH 3T3 fibroblasts^39^. Although GDF15 was used at a 25-fold higher concentration than TGF-β, its effect on fibroblast growth was much less than that of TGF-β. This suggests that GDF15 acts as a partial agonist to the TGF-β receptor. In general, weak agonists can stimulate receptors in the absence of full agonists, but function to inactivate receptor signaling in the presence of full agonists because it competes with the agonist for binding to receptors^40^. Considering the TGF-β-enriched environment in fibrotic lungs^40^. GDF15 could act as an inhibitor of fibrogenesis. However, the precise role of GDF15 in lung fibroblast growth and activation remains to be investigated.

In conclusion, cytokines CST3 and GDF15 are secreted from normal epithelial cells and inhibit growth and activation of lung fibroblasts. These cytokines are severely deregulated in mouse and human lungs undergoing fibrosis, and systemic administration of them preserves the air way architecture by lessening collagen deposition in the interstitium of bleomycin-treated mouse lung. CST3 and GDF15 appear to be bona fide regulators that prevent excessive proliferation and activation of fibroblasts in injured lungs.

## Materials and methods

### Cell culture and conditioned media

CCD-18Lu (normal lung fibroblast), A549 (adenocarcinomic alveolar epithelial cell), and HCT116 (colon cancer cell) were purchased from the American Type Culture Collection (ATCC, Manassas, VA); hPAE (human pulmonary alveolar epithelial cell) was from ScienceCell Research Laboratory (Carlsbad, CA). hPAE cells were cultured in Eagle Alveolar Epithelial Cell Medium supplied by the manufacturer. To prepare conditioned media, various types of cells were seeded in a 100-mm dish at 80% confluency and incubated in cell type-specific media for 24 h. Next day, the cells were washed with PBS and incubated commonly in Dulbecco’s Modified Eagles media without FBS. After 2 or 3 day-incubation (~1 × 10^7^ cells per 100-mm dish), the CM (5 mL per dish) was centrifuged, filtered, and mixed with an equal volume of a fresh medium.

### Human lung tissues

Lung biopsy samples were obtained from 10 patients with ILD and 10 lung cancer patients without ILD. Of 10 ILD patients, 3 lung cancer patients with interstitial pneumonia were included. ILD was finally diagnosed by pathologists in Seoul National University Hospital. Detailed information on tissue donors is summarized in Supplementary table 1. The study protocol was approved by the Institutional Review Board of Seoul National University Hospital (approval No. 1704-172-849).

### Bleomycin-induced pulmonary fibrosis

C57BL/6J mice (male, 11 weeks) were purchased from Central Laboratory Animal Inc., and kept in specific pathogen-free rooms. Mice were anesthetized with a mixture of tiletamine/zolazepam (30 mg/kg) and xylazine (10 mg/kg), and subjected to an intratracheal injection with saline or bleomycin sulfate (2 mg/kg, MBcell, CA) in a total volume of 50 μL. CST3/GDF15 peptides (50 μg/kg each) or PBS were injected intraperitoneally into mice on days 6, 9, 12, 15, 18 after the bleomycin challenge. Recombinant peptides of mature CST3 (aa. 27–146, NM_000099) and mature GDF15 (aa. 195–308, NM_004864) were purchased from Abcam and Sino Biological Inc. (Beijing, China), respectively. On day 21, the lung tissues were removed from mice and divided into two lobes. The left lobes were fixed in 4% paraformaldehyde, and the right lobes were stored in liquid nitrogen. All procedures were approved by the Seoul National University Institutional Animal Care and Use Committees (Approval No. 141006-3).

### Mouse lung fibroblast isolation and culture

Normal lung fibroblasts were isolated from the lung tissues of normal mice. To get pulmonary fibrosis-associated fibroblasts (PFAF), mice were subjected to a single challenge of bleomycin via bronchial instillation and the lungs were excised on day 12. Lung tissues were washed and dissected using surgical blades. The minced tissues were digested with 2.4 U/mL dispase and 0.1% collagenase at 37 °C for 45 min. The suspension was filtered through 70 μm nylon strainer and centrifuged at 1000 × *g* for 10 min. The cell pellet was resuspended in DMEM containing 20% FBS and incubated overnight at 37 °C. Attached fibroblasts were subcultured in DMEM containing 10% FBS, 100 U/mL penicillin, and 100 g/mL streptomycin.

### Cytokine profiling

Cytokine profiling was performed using the proteome profiler Human XL cytokine array kit (# ARY022) provided by R&D Systems. Cells were incubated in serum-free media for 3 days and the conditioned media were prepared as mentioned above. The media (1 mL per membrane) were applied to the nitrocellulose-based array membrane and incubated at 4 °C overnight. Array membranes were treated with the detection antibody cocktail (R&D Systems) for 1 h and further with the streptavidin-HRP solution for 30 min. The immune complexes were visualized using the Chemi-Reagent Mix kit and the arrays were exposed to X-ray films. Based on the mean intensity of reference spots (A1/2, A23/24, and J1/2), the intensity of each dot was normalized.

### Masson’s trichrome assay and Ashcroft scoring

The paraffin sections (4 μm) of lungs were deparaffinized, rehydrated, and sequentially stained with Weigert’s iron hematoxylin and biebrich scarlet-acid fuchsin for 10 min. Finally, the sections were stained with 2.5% aniline blue for 10 min and destained with 1% glacial acetic acid for 3 min. Photomicrographs were taken from four random fields in a tissue section. The severity (0 to 8) of interstitial fibrosis was evaluated based on the Ashcroft fibrosis scoring system^41^.

### Hydroxyproline assay

The frozen right lungs were homogenized in distilled water (100 μL/10 mg tissue). The homogenates were incubated at 95 °C for 3 h and centrifuged at 16,000 × *g* for 10 min. Collagen content in the supernatant was measured using the hydroxyproline colorimetric assay kit provided by BioVision (Milpitas, CA). Hydroxyproline levels were spectrophotometrically determined based on the absorbance at 560 nm.

### Immunoblotting

Cells were lysed in a denaturing SDS sample buffer. Proteins in the cell lysates were separated on SDS/10-15% polyacrylamide gels and transferred to Immobilon-P membranes. The membranes were pre-incubated with 5% skim milk for 30 min, incubated overnight with primary antibodies (1:1000 dilution) at 4 °C, and incubated with HRP–conjugated secondary antibodies (1:5000) at room temperature for 1 h. The immune complexes were visualized using the SuperSignal West Femto kit (Thermo Scientific, Waltham, MA). Antibodies against Smad2, p-Smad2, Smad3, and p-Smad3 were purchased from Cell Signaling Tech. (Danvers, MA); TGFβR1 antibody from Santa Cruz Biotech. (Dallas, TX); α-SMA, collagen-1α, and p-TGFβR1 antibodies from Abcam (Cambridge, MA); CST3 antibody from R&D Systems (Minneapolis, MN); GDF15 antibody from Fisher Scientific (Pittsburgh, PA).

### Immunohistochemistry and immunofluorescence

Paraffin sections (4 μm) of lung tissues were deparaffinized, rehydrated, and autoclaved at 121 °C for 10 min in 100 mM citrate buffer (pH 6.0) to retrieve antigens. After treated with 3% hydrogen peroxide for 10 min, the sections were incubated in 10% bovine serum at room temperature for 1 h to block nonspecific interactions. They were incubated with an antibody against collagen-1α (1:250 dilution; Abcam), α-SMA (1:500; Abcam), CST3 (1:1000; R&D Systems), or GDF15 (1:10,000; Abcam) overnight at 4 °C. For immunohistochemistry, the sections were incubated with biotinylated secondary antibodies (1:500) provided by Vector Laboratories (Burlingame, CA). The immune complexes were visualized using the VECTASTAIN ABC Kit (Vector laboratories). For immunofluorescence, the sections were incubated with Alexa Fluor 488-conjugated anti-goat secondary antibody (Thermo Fisher), and counterstained with DAPI (Invitrogen). Fluorescent images were acquired using Olympus fluorescence microscope (DP30BW, Melville, NY) or Nikon confocal laser microscopy (A1, Nikon Instruments, Tokyo, Japan). Four high power fields were randomly selected in each section to analyze stained areas or fluorescent intensities using the ImageJ program (NIH, Bethesda, MD).

### Quantitative RT-PCR

Total RNAs were extracted from cells using the TRIzol kit (Thermo Fisher Scientific). cDNAs were synthesized from 2 μg of RNAs using M-MLV reverse transcriptase (Promega, San Luis Obispo, CA). The cDNAs were amplified with SYBR Green (Enzynomics, Daejeon, Korea), and quantified using a 7900HT real-time PCR detection system (Bio-Rad, Hercules, CA). The sequences of PCR primers are informed on Supplementary Method.

### Statistical analysis

The statistical analyses were performed using the SPSS (window version 15.0.0) software package. Comparisons between two groups were analyzed by Student’s *t*-test for parametric data or by Mann–Whitney *U* test for non-parametric data. We performed one-way ANOVA and post hoc Tukey test for analyzing three or more groups. Survival rates were analyzed by the log-rank test. Significant difference was defined when *P* < 0.05.

## Electronic supplementary material

Supplemental figures
