# Supplementary material for: Epithelial cell-derived cytokines CST3 and GDF15 as potential therapeutics for pulmonary fibrosis
Source: Cell Death Dis. 2018 May 3;9(5):506. doi: 10.1038/s41419-018-0530-0 (PMC5938700; doi:10.1038/s41419-018-0530-0)
Supplement: Supplementary file 1 — Supplemental figures [file 41419_2018_530_MOESM1_ESM.pdf]

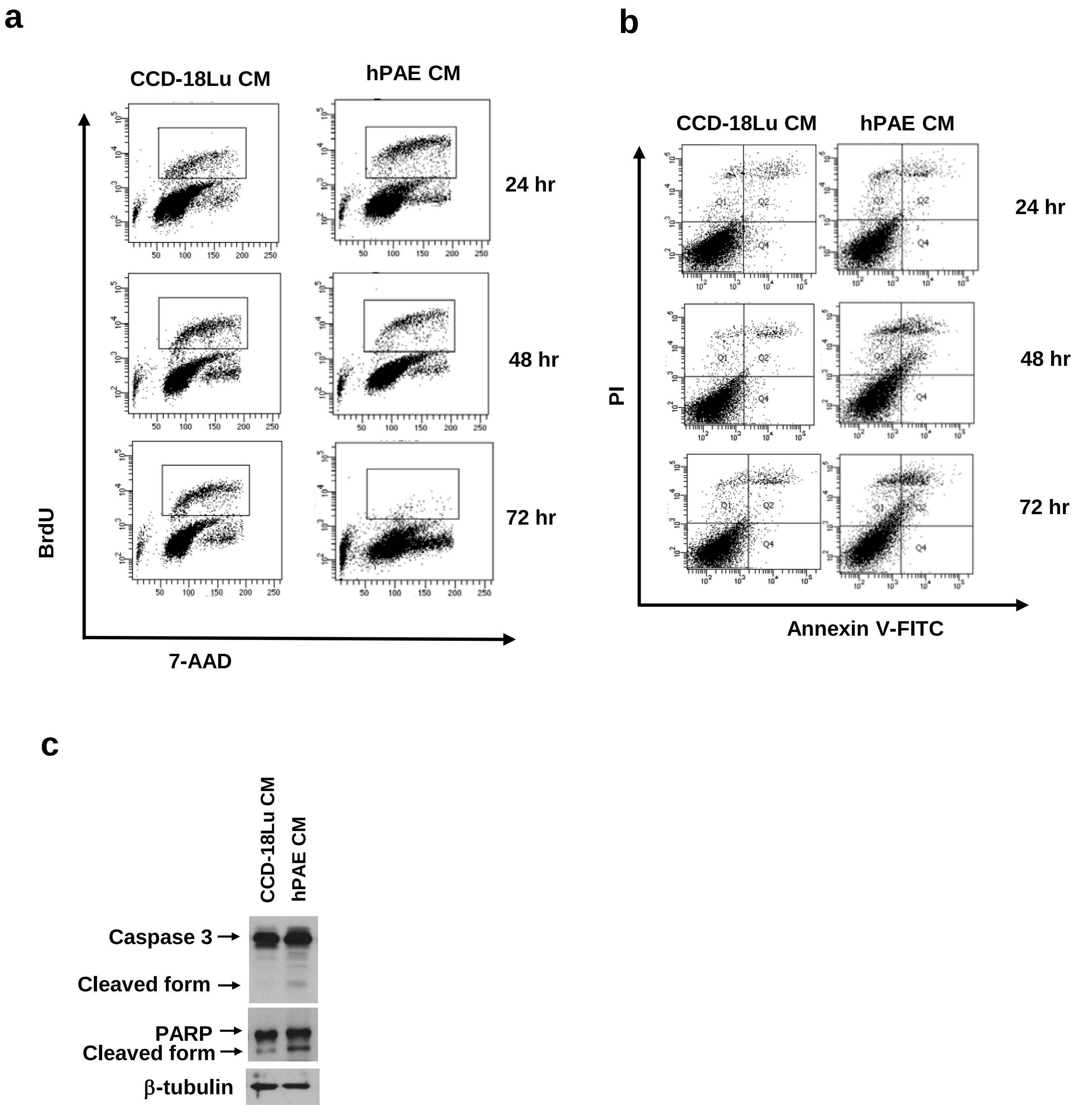

Supplementary Figure S1. Analyses for cell proliferation and death. CCD-18Lu cells were incubated for 24 hours, 48 hours, or 72 hours in the 72 hour-conditioned media from CCD-18Lu and hPAE cells. (a) CCD-18Lu cells were co-stained with BrdU and 7-AAD, and analyzed by flow cytometry. (b) CCD-18Lu cells were co-stained with annexin-V and propodium iodine, and analyzed by flow cytometry. (c) Caspase 3 and PARP expression level were determined by western blot. Each panel is representative of three independent experiments.

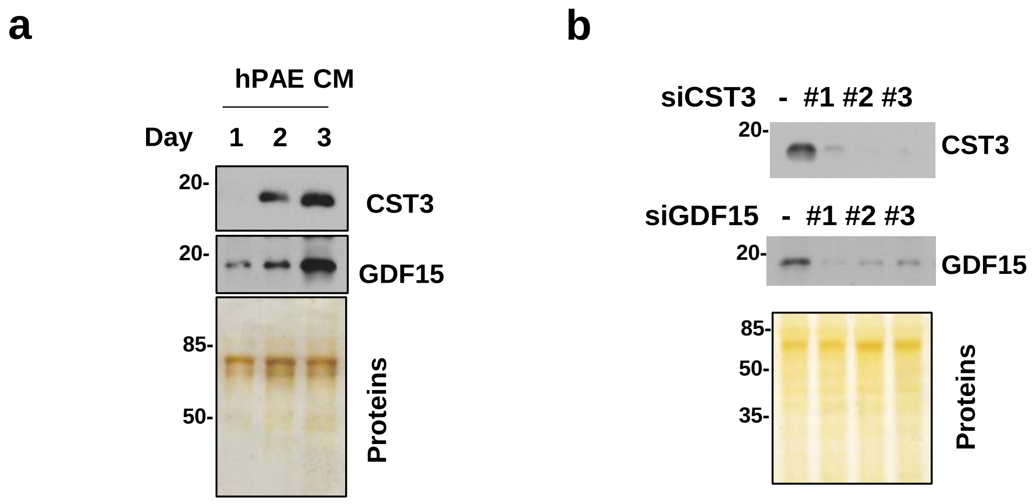

Supplementary Figure S2. CST3 and GDF15 levels in hPAE conditioned media. (a) Conditioned media (CMs) were collected from hPAE on the indicated times and proteins were precipitated using TCA. (b) CMs were collected on day 3 from hPAE which had been transfected with the indicated siRNAs. Proteins were precipitated using TCA. Proteins were subjected to immunoblotting and silver staining.

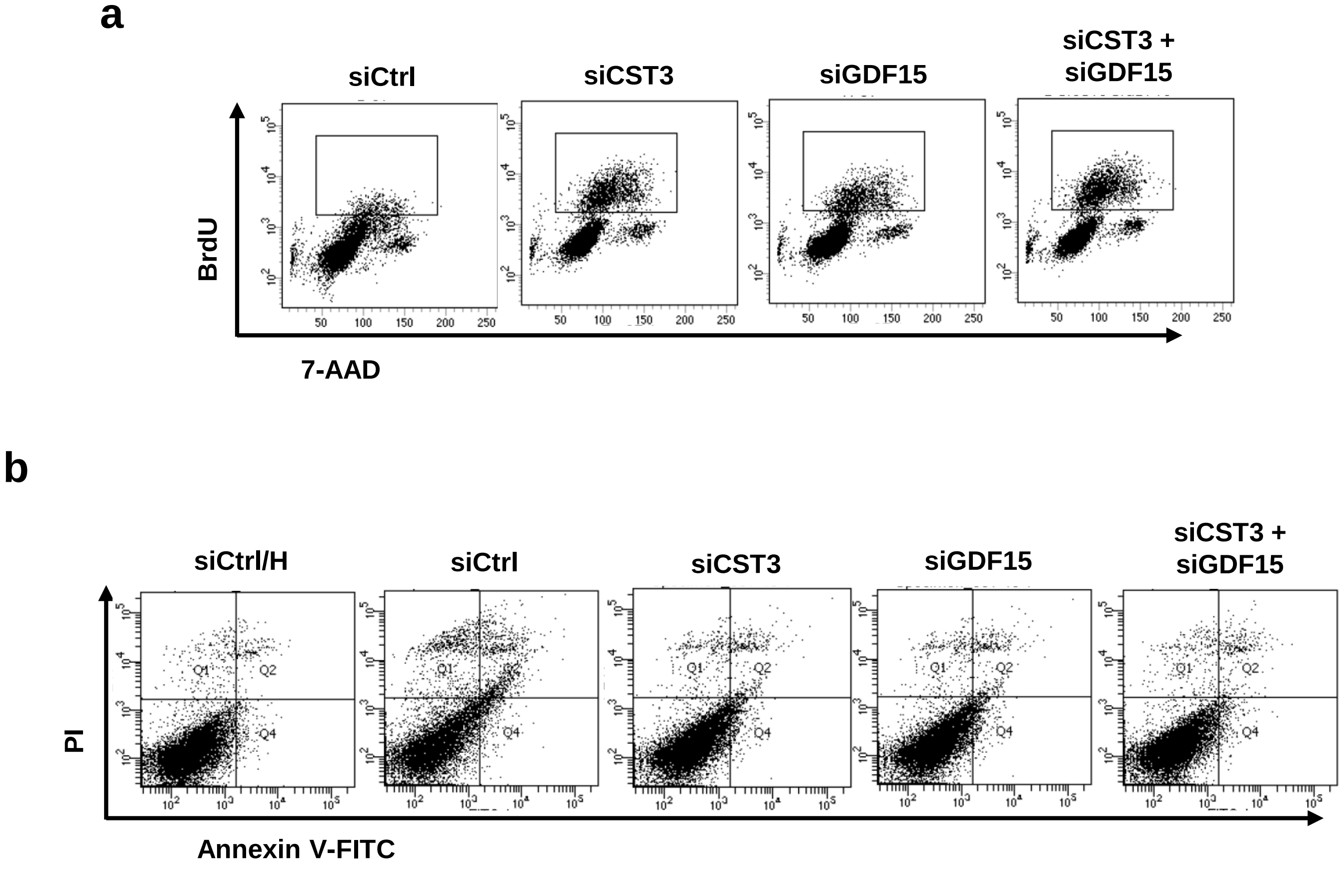

Supplementary Figure S3. Effects of CST3 and GDF15 knock-down on fibroblast proliferation and death. hPAE cell were transfected with each or both of CST3- and GDF15-targeting siRNAs (20 nM of each). CCD-18Lu cells were incubated for 48 hours in the 72 hour-conditioned media from the transfected cells. (a) Cells were co-stained with BrdU and 7-AAD to analyze cell proliferation. (b) Cells were co-stained with annexin-V and propodium iodine to analyze apoptosis and necrosis. Each panel shows a representative flow plot of three experiments.

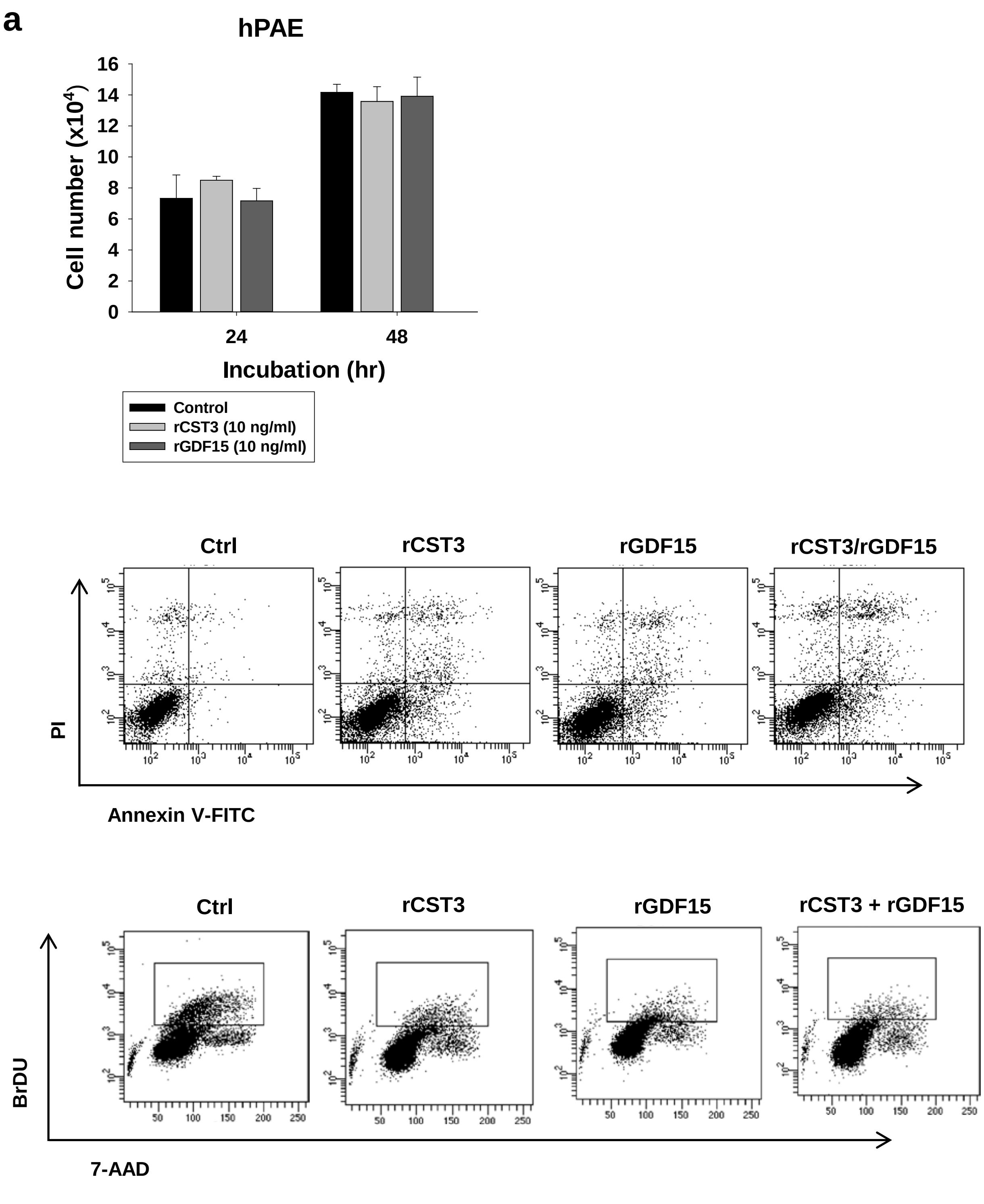

Supplementary Figure S4. Effects of recombinant peptides on cell growth in hPAE. (a) hPAE cells were treated with a recombinant peptide of CST3 or GDF15 for 24 hours or 48 hours, and the numbers of viable cells were counted using a hemocytometer. Each bar represents the mean and s.d. from four independent experiments. (b) PFAF cells were treated by with a recombinant peptide of CST3 or GDF15 for 24 hours, after that cells were stained by annexin V and PI. (c) Both CST3 or GDF15 treated PFAF cells were stained by BrdU and 7-AAD after 24 hours.

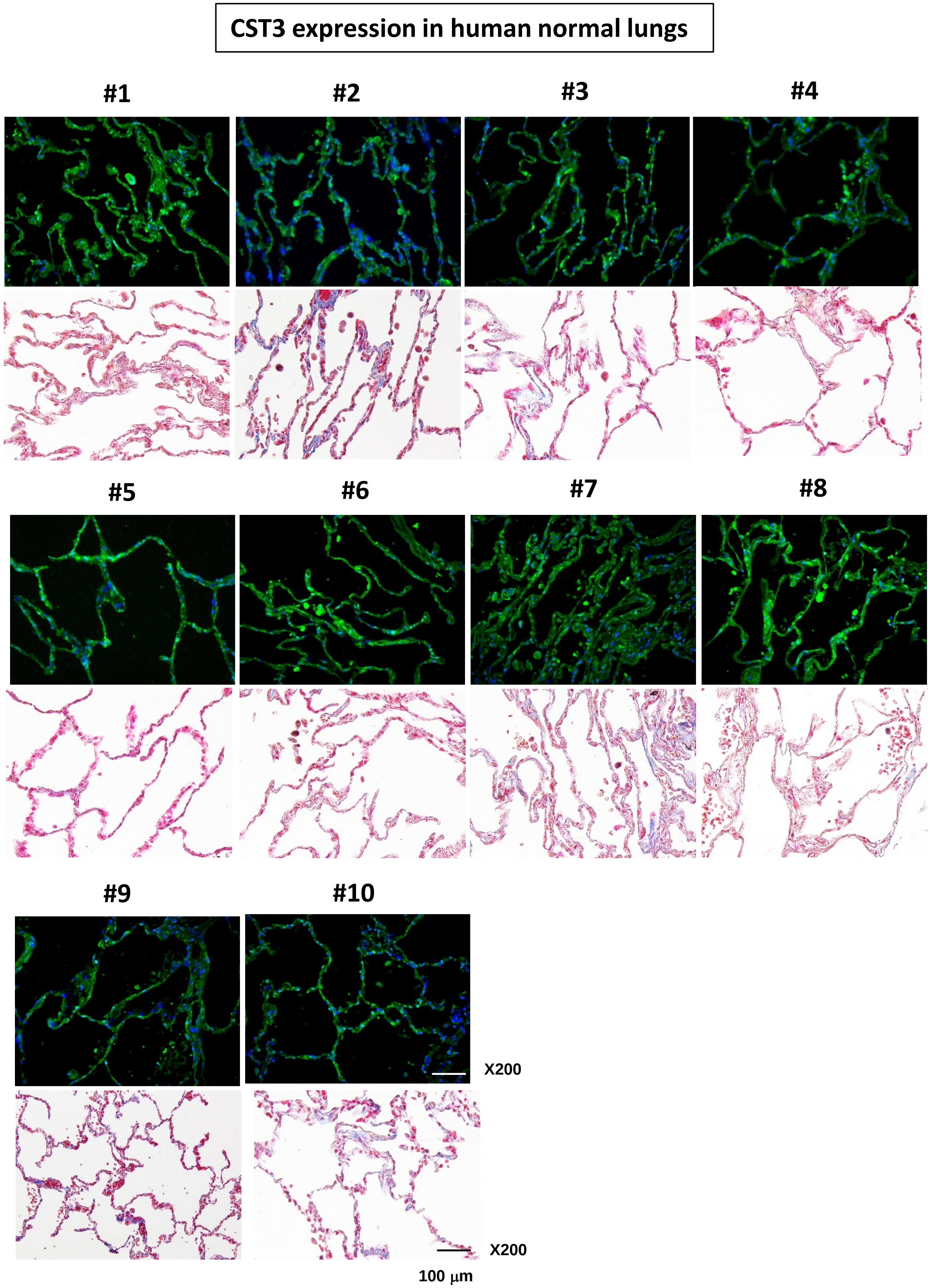

Supplementary Figure S5. Expression of CST3 in normal lung tissues from 10 patients with adenocarcinoma. Lung tissue specimens were subjected to immunofluorescence analysis using anti-CST3 antibody or stained with Masson's trichrome.

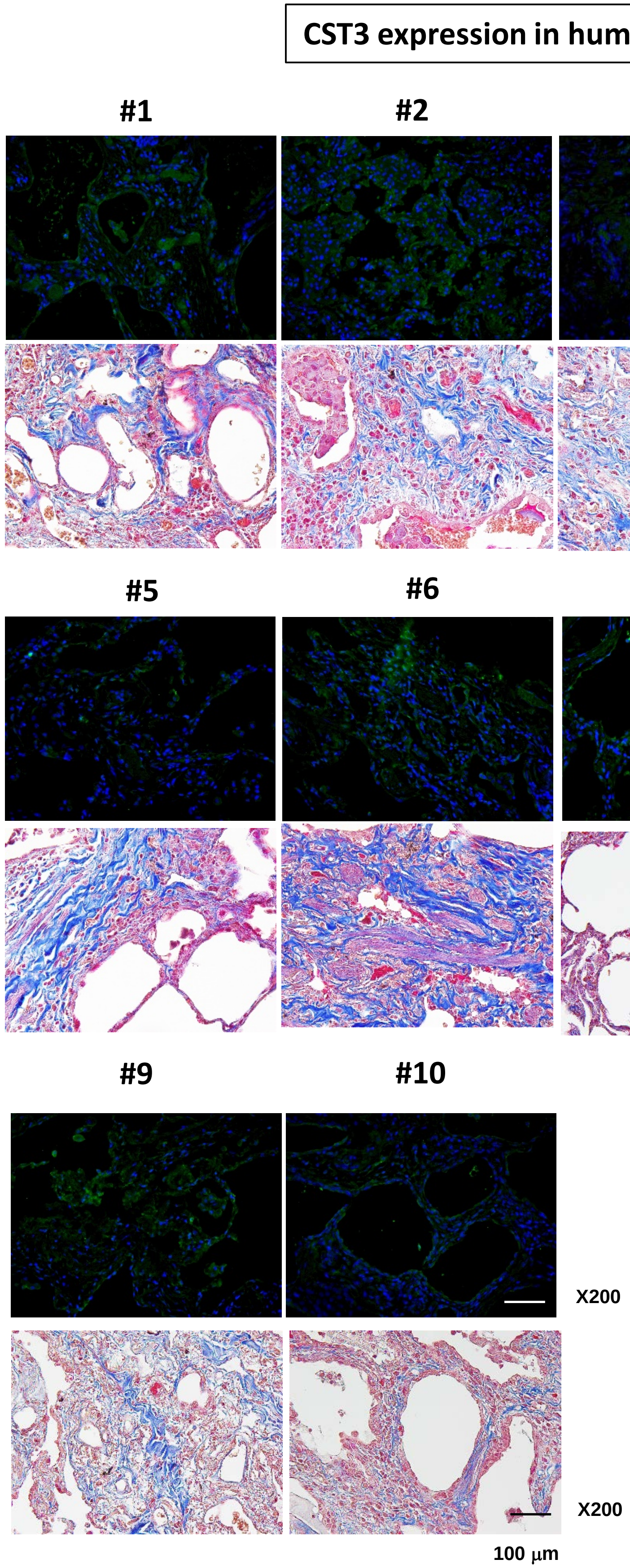

Supplementary Figure S6. Expression of CST3 in fibrotic lung tissues from 10 patients with interstitial lung disease. Lung tissue specimens were subjected to immunofluorescence analysis using anti-CST3 antibody or stained with Masson's trichrome.

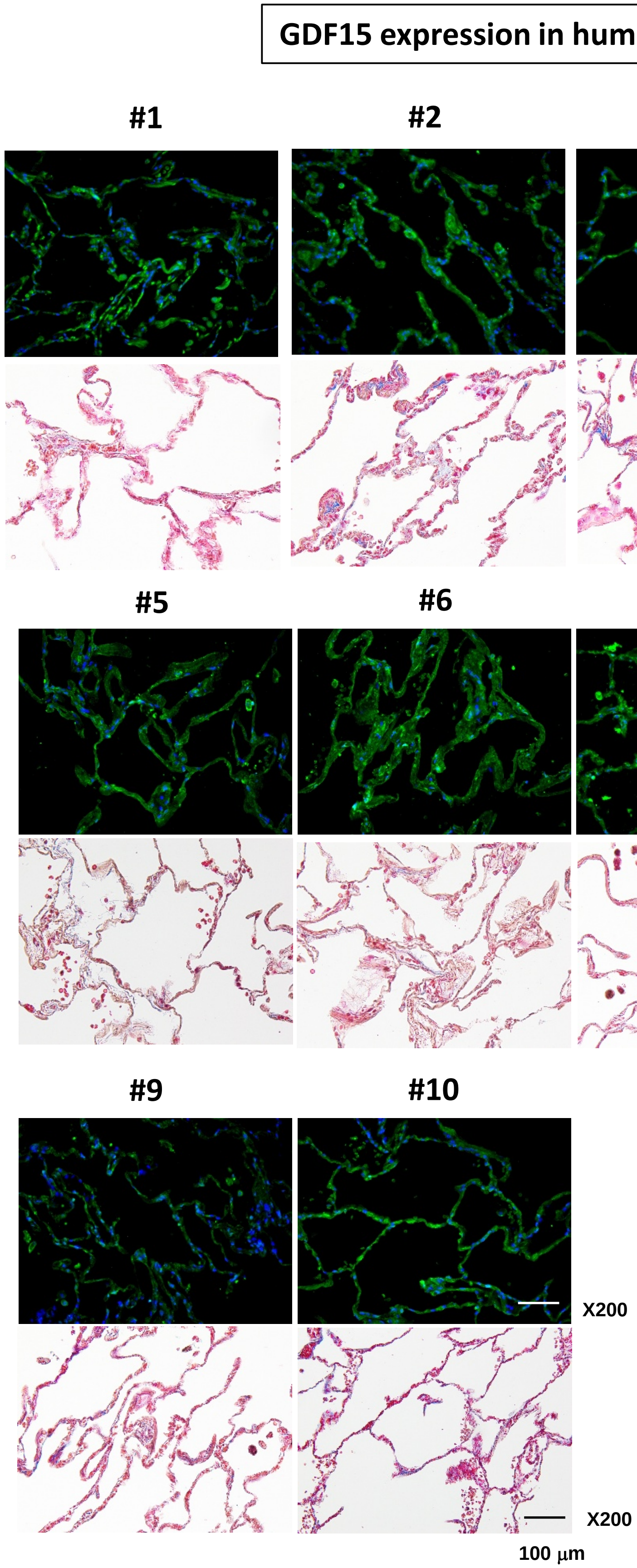

Supplementary Figure S7. Expression of GDF15 in normal lung tissues from 10 patients with adenocarcinoma. Lung tissue specimens were subjected to immunofluorescence analysis using anti-GDF15 antibody or stained with Masson's trichrome.

GDF15 expression in human ILD lungs

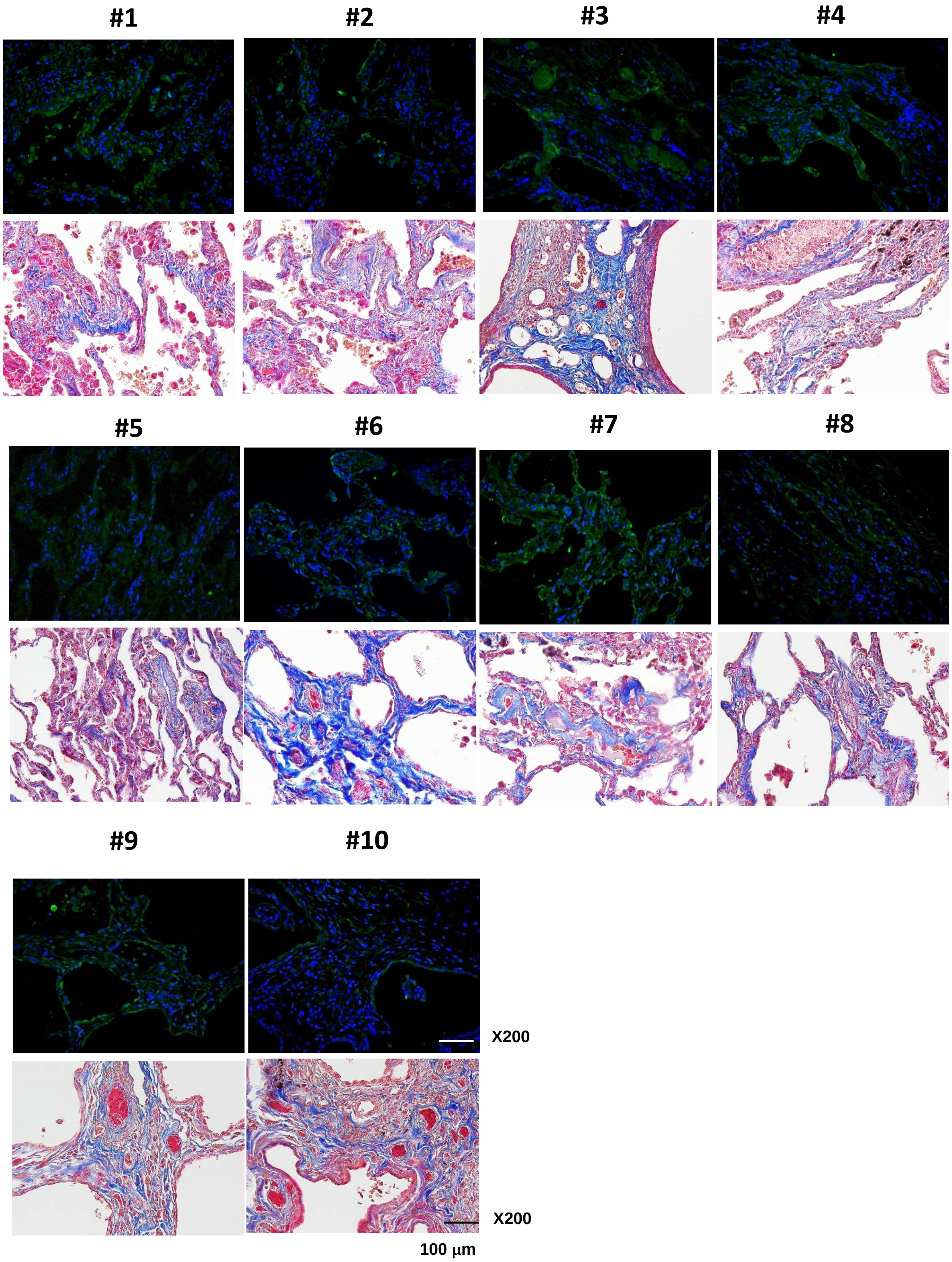

Supplementary Figure S8. Expression of GDF15 in fibrotic lung tissues from 10 patients with interstitial lung disease. Lung tissue specimens were subjected to immunofluorescence analysis using anti-GDF15 antibody or stained with Masson's trichrome.

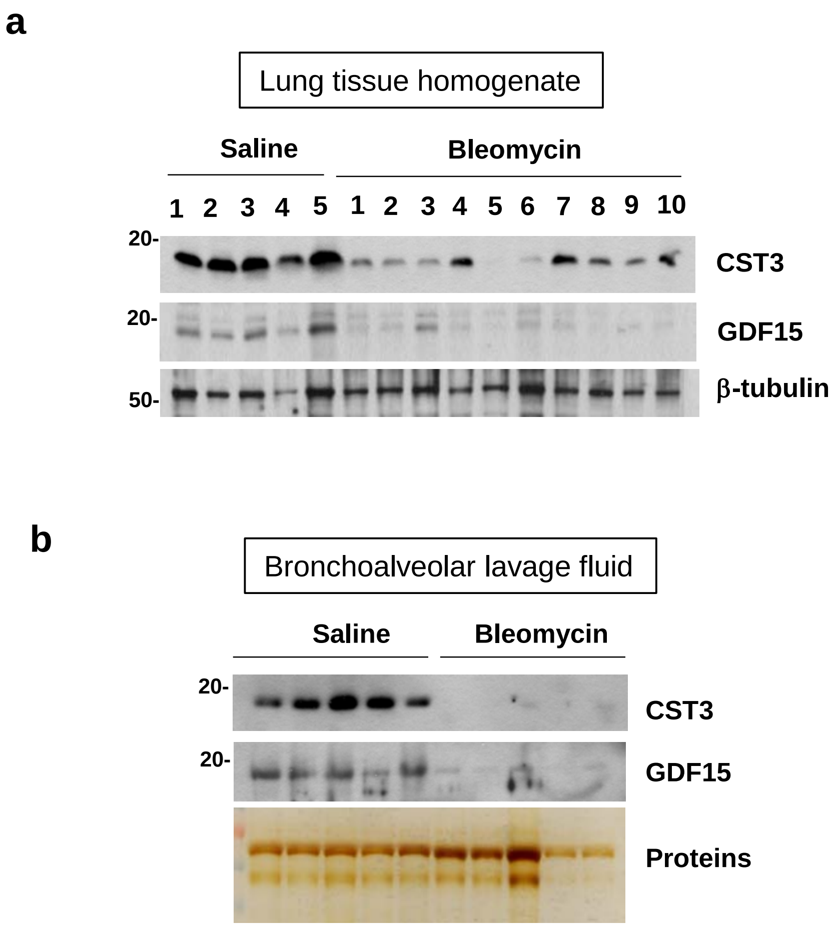

Supplementary Figure S9. CST3 and GDF15 levels in lung tissues and BALFs. (a) Western blot analysis of CST3 and GDF15 proteins from saline administrated mouse lung homogenate (saline) and bleomycin administrated for 21 days mouse lung homogenate using antibodies to the indicated proteins. (b) Bronchoalveolar lavage fluid were collected from saline or bleomicin treated mouse for 21 days, and protein in BALF were precipitated by TCA. CST3 and GDF15 cytokine level in BALF were detected by western blotting.

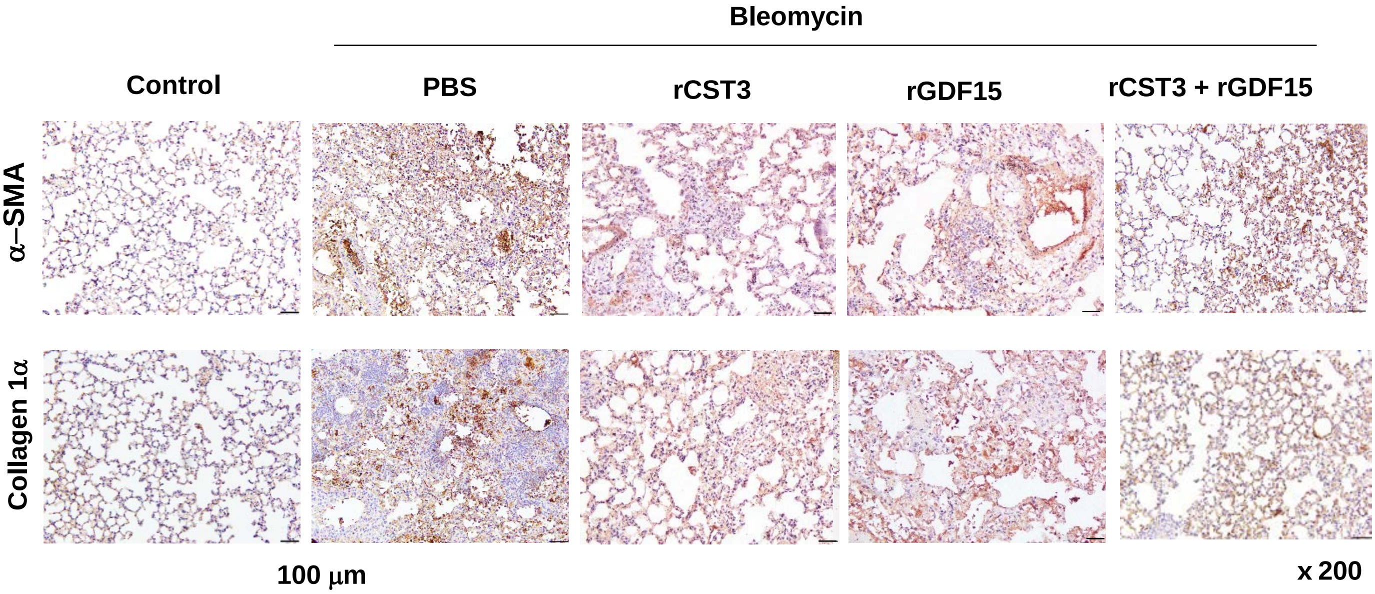

Supplementary Figure S10. α-SMA and collagen-1α in lung tissues were immunohistochemically stained.
